# Combining immune-related adverse events and inflammatory profiles enhances prognostic accuracy in metastatic melanoma under PD-1-based therapy

**DOI:** 10.3389/fimmu.2025.1683533

**Published:** 2025-10-01

**Authors:** Dionysios Garmpis, Guillermo Hidalgo-Gadea, Cornelia Mauch, Julia K. Tietze, Cindy Franklin

**Affiliations:** ^1^ Department of Dermatology and Venereology, University of Cologne, Faculty of Medicine and University Hospital Cologne, Cologne, Germany; ^2^ Department of Biopsychology, Faculty of Psychology, Ruhr University Bochum, Bochum, Germany; ^3^ Department of Dermatology and Venereology, Ruhr University Bochum, Bochum, Germany; ^4^ Department of Dermatology and Venereology, University Hospital Rostock, Rostock, Germany

**Keywords:** immune checkpoint inhibitors, melanoma, immune-related adverse events (irAEs), biomarkers, C-reactive protein (CRP), neutrophil-to-lymphocyte ratio (NLR), progression-free survival, overall survival

## Abstract

**Background:**

Immune checkpoint inhibitors (ICIs) have improved outcomes in advanced melanoma, yet predictive biomarkers for treatment response and survival remain limited. Immune-related adverse events (irAEs) are frequent during ICI therapy and have been associated with improved outcomes, while baseline inflammatory markers—such as C-Reactive protein (CRP) and neutrophil-to-lymphocyte ratio (NLR)—often predict poor prognosis. However, no study to date has systematically integrated irAE characteristics and blood-based inflammation profiles to evaluate their combined prognostic value across different therapy lines.

**Methods:**

We retrospectively analyzed 231 patients with unresectable stage IIIC–IV melanoma treated with PD-1-based ICIs at the University Hospital Cologne (2015–2021). Patients were stratified into first-line (n=149) and higher-line (n=82) groups. We assessed the occurrence, number, type, and severity of organ-specific and non-specific irAEs, and correlated these with progression-free survival (PFS) and overall survival (OS) alongside baseline hematological markers (CRP, neutrophils, lymphocytes, lymphocyte-to-monocyte ratio (LMR), NLR) using multivariate Cox regression.

**Results:**

Across both therapy lines, the occurrence, higher number, and moderate severity (CTCAE I–III) of organ-specific irAEs independently predicted longer PFS and OS, whereas high-grade irAEs (≥IV) were associated with worse OS. In first-line therapy, ≥2 irAEs conferred markedly prolonged PFS (HR 0.49; *p*=0.007) and OS (HR 0.53; *p*=0.040). Elevated CRP and neutrophils predicted shorter survival, while higher lymphocyte counts and LMR were favorable; CRP emerged as the most consistent independent prognostic biomarker. Eosinophil counts predicted both irAE development and improved survival in univariate analyses only. Combining irAEs with CRP and lymphocyte-based markers improved PFS prediction, particularly in first-line therapy.

**Conclusion:**

Integrating irAE characteristics with baseline inflammatory biomarkers enhances prognostic stratification in ICI-treated melanoma, especially in first-line settings. Moderate irAEs appear to reflect beneficial immune activation, whereas high-grade events may compromise outcomes. CRP and lymphocyte-based indices provide additive value and should be considered in future biomarker-driven patient selection and monitoring strategies.

## 1 Introduction

Immune checkpoint inhibitors (ICIs) have markedly improved the prognosis of patients with advanced melanoma by restoring antitumor immune responses (1). Agents targeting CTLA-4 (e.g., ipilimumab) or PD-1 (e.g., nivolumab, pembrolizumab), alone or in combination, are now standard treatments across all disease stages (1). A PD-1/LAG-3-directed antibody combination has also been approved more recently, though it is not yet available in Germany. Despite these advances, a substantial proportion of patients derive limited benefit, and reliable predictive biomarkers remain scarce (2). ICIs frequently induce immune-related adverse events (irAEs), which affect up to 95% of patients and reflect systemic immune activation (3, 4). These toxicities can involve virtually any organ system and vary in frequency and severity depending on the therapeutic regimen (4–6). While irAEs pose clinical challenges, mounting evidence suggests they may also reflect effective immune activation and be associated with improved survival, particularly in melanoma (7–9). However, it remains unclear how specific irAE characteristics (e.g., type, number, and severity) influence outcomes such as progression-free survival (PFS) and overall survival (OS), and whether these associations differ between first- and later-line therapies (8, 9). Baseline inflammatory blood markers, including CRP, neutrophil and lymphocyte counts, and derived indices like the neutrophil-to-lymphocyte ratio (NLR), have also been associated with outcomes in ICI-treated patients (10). Yet, the interplay between these parameters and irAE development, and how this interaction influences survival, has not been systematically investigated. This study addresses this gap by integrating irAE profiles and baseline blood-based inflammatory markers into multivariate prognostic models of metastatic melanoma patients, stratified by therapy line. To our knowledge, this is the first analysis to combine both domains in a unified framework, aiming to refine risk stratification and inform personalized immunotherapy strategies. While observational in design, our findings are of high clinical relevance and may serve as a foundation for future translational efforts to better understand the immune biology underlying ICI response and toxicity.

## 2 Materials and methods

### 2.1 Study design

**Figure 1:**
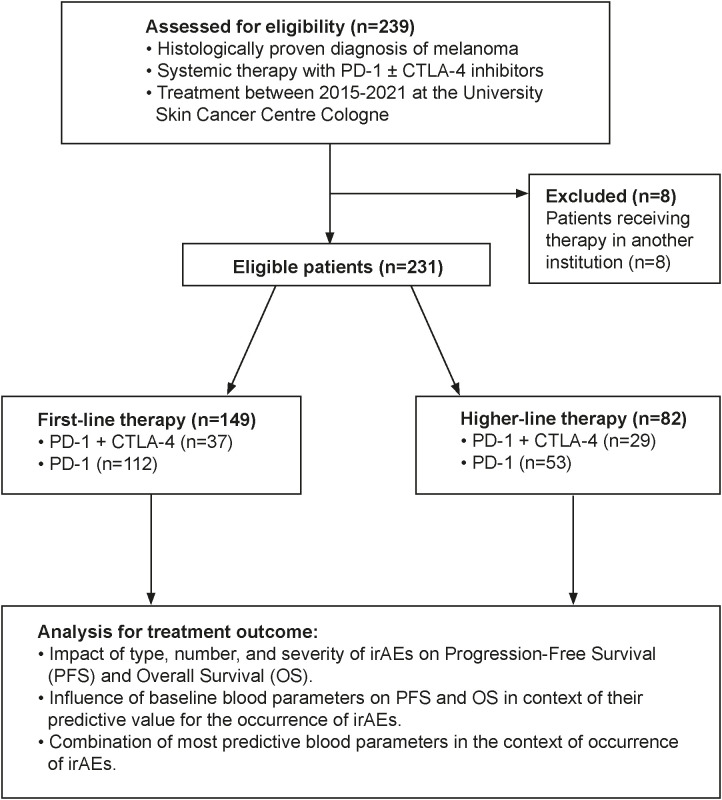
Study flow.

This retrospective monocentric cohort study included 231 patients with histologically confirmed, unresectable or metastatic stage IIIC–IV melanoma (AJCC v8) treated with immune checkpoint inhibitors (ICIs) at the University Hospital Cologne between 2015 and 2021. All patients received either PD-1 monotherapy (nivolumab/pembrolizumab) or a combination of nivolumab and ipilimumab. Baseline blood samples were obtained within one week prior to initiation of either first-line or higher-line ICI therapy. Data were collected and analyzed retrospectively. Exclusion criteria comprised initiation of systemic ICI treatment outside the University Hospital of Cologne, absence of baseline blood samples within one week prior to therapy start, incomplete clinical follow-up data, or transfer to another institution shortly after treatment initiation. Eight patients were excluded due to transfer to another institution, leaving 149 patients in the first-line and 82 in the higher-line therapy groups (**Figure 1**).

### 2.2 Patient and treatment characteristics

Baseline clinical and laboratory data were recorded prior to treatment initiation. Clinical parameters included age, sex, ECOG performance status, TNM classification (AJCC stage), presence of organ metastases, and BRAF mutation status. Information on prior or concomitant corticosteroid use was recorded, including both immunosuppressive treatment of immune-related adverse events (irAEs) and pre-treatment corticosteroids given for other indications (e.g., cerebral edema due to brain metastases). Blood-based biomarkers were obtained from samples taken within one week before the start of first-line or higher-line ICI treatment at the University Hospital Cologne. These included absolute and relative counts of leukocytes, lymphocytes, neutrophils, eosinophils, monocytes, and platelets, as well as serum levels of C-reactive protein (CRP), lactate dehydrogenase (LDH), and S100B. Derived inflammatory indices such as the neutrophil-to-lymphocyte ratio (NLR), lymphocyte-to-monocyte ratio (LMR), and platelet-to-lymphocyte ratio (PLR) were calculated. Adverse events were documented during follow-up visits and categorized as organ-specific irAEs (e.g., cutaneous, gastrointestinal, endocrine) or non-specific (e.g., fatigue, fever). IrAEs were graded according to CTCAE v6.0. For survival analyses immune-related adverse events (irAEs) were categorized into three groups: CTCAE grade 0, I–III and ≥ grade IV. Grades I–III were considered mild to moderate, typically manageable with supportive measures or temporary treatment interruption, whereas grade IV events were regarded as life-threatening and clinically distinct. This grouping was chosen to ensure sufficient statistical power and comparability. IrAEs were treated per institutional guidelines. Radiological response assessments were performed every 3 months. An ethics approval for the retrospective analysis of pseudonymized clinical data has been obtained from the Ethics Committee of the Medical Faculty, University of Cologne.

### 2.3 Statistical analysis

**Figure 2:**
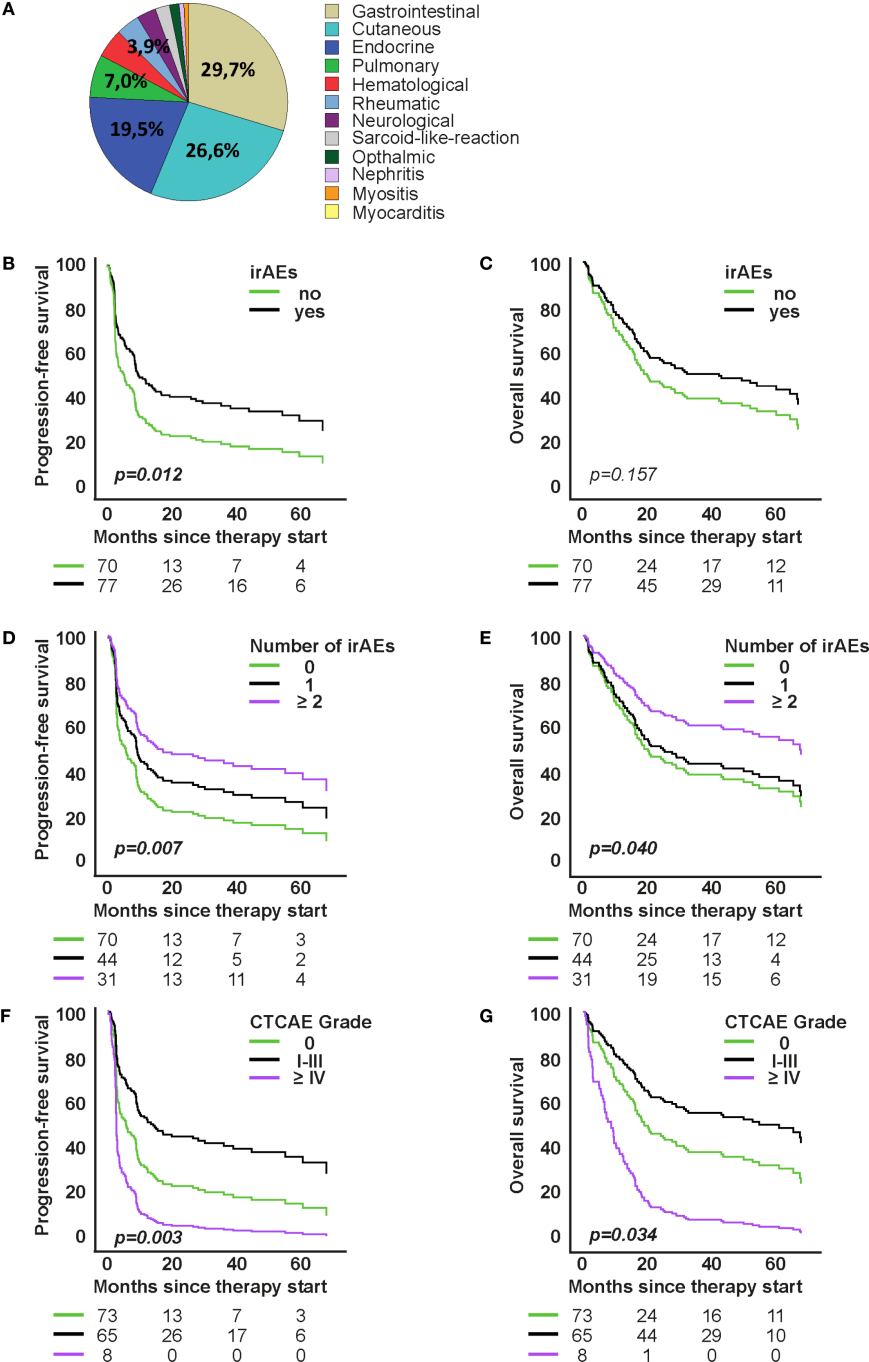
Association of organ-specific immune-related adverse events (irAEs) with survival under first-line immune checkpoint inhibitor therapy. **(A)** Distribution of organ-specific irAEs. **(B, C)** Model-based survival curves from Cox proportional hazards models for progression-free survival (PFS) and overall survival (OS) by irAE occurrence **(D–G)**: Cox models for PFS and OS by number of organ-specific irAEs and by toxicity grade. Curves show model-based survival functions from the respective Cox models. Hazard ratios (HR) and p-values are from the Wald test of the model coefficients.

**Figure 3:**
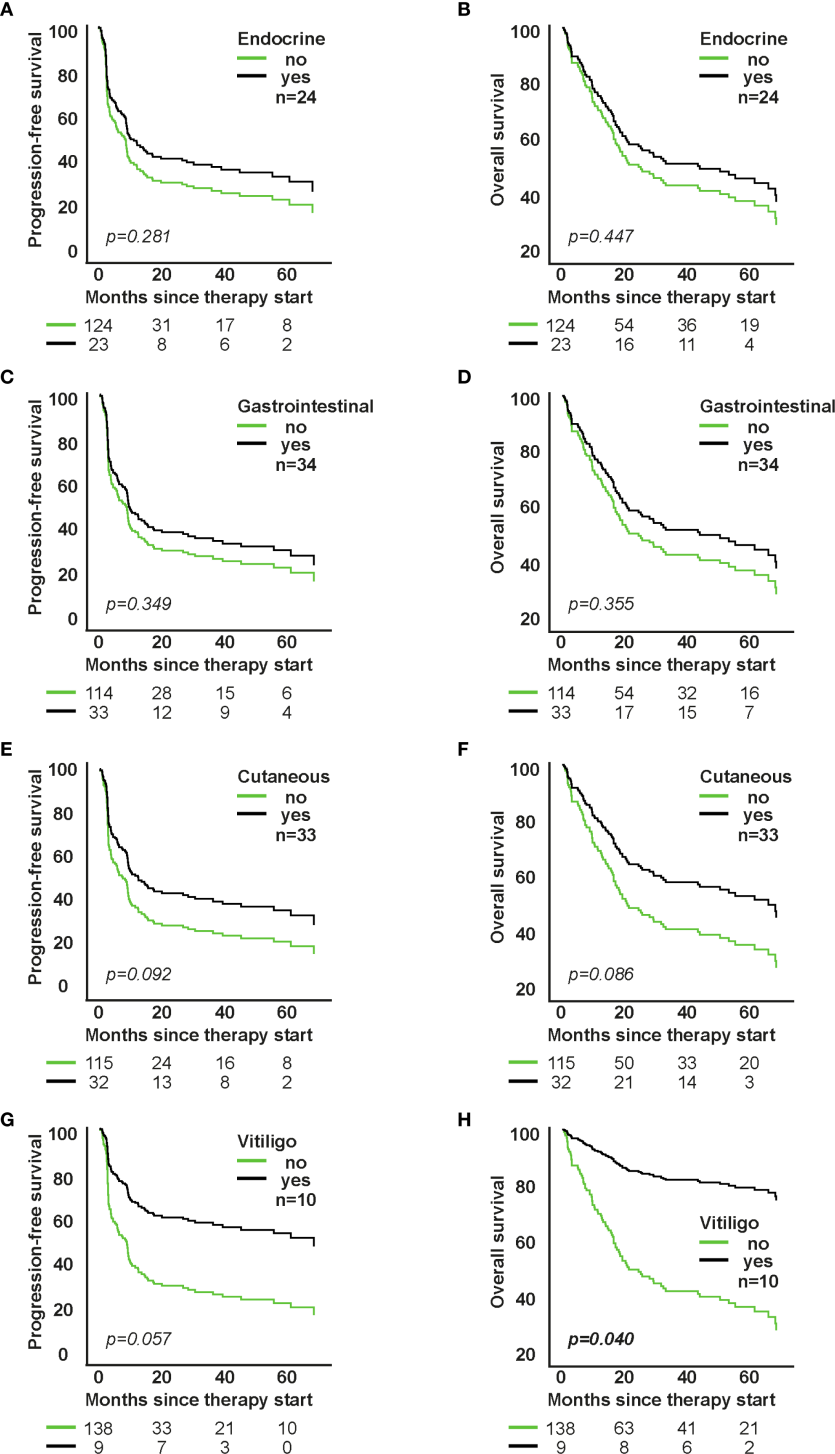
Impact of individual organ-specific irAE types on survival in patients receiving first-line immune checkpoint inhibitor therapy. **A**, **C**, **E**, **G**: PFS; **B**, **D**, **F**, **H**: OS. Each panel shows model-based survival curves from a separate Cox proportional hazards model fitted for the respective irAE type (e.g., cutaneous, gastrointestinal, endocrine, pulmonary). HRs (95% CI) and p-values are from the Wald test of the model coefficients.

**Figure 4:**
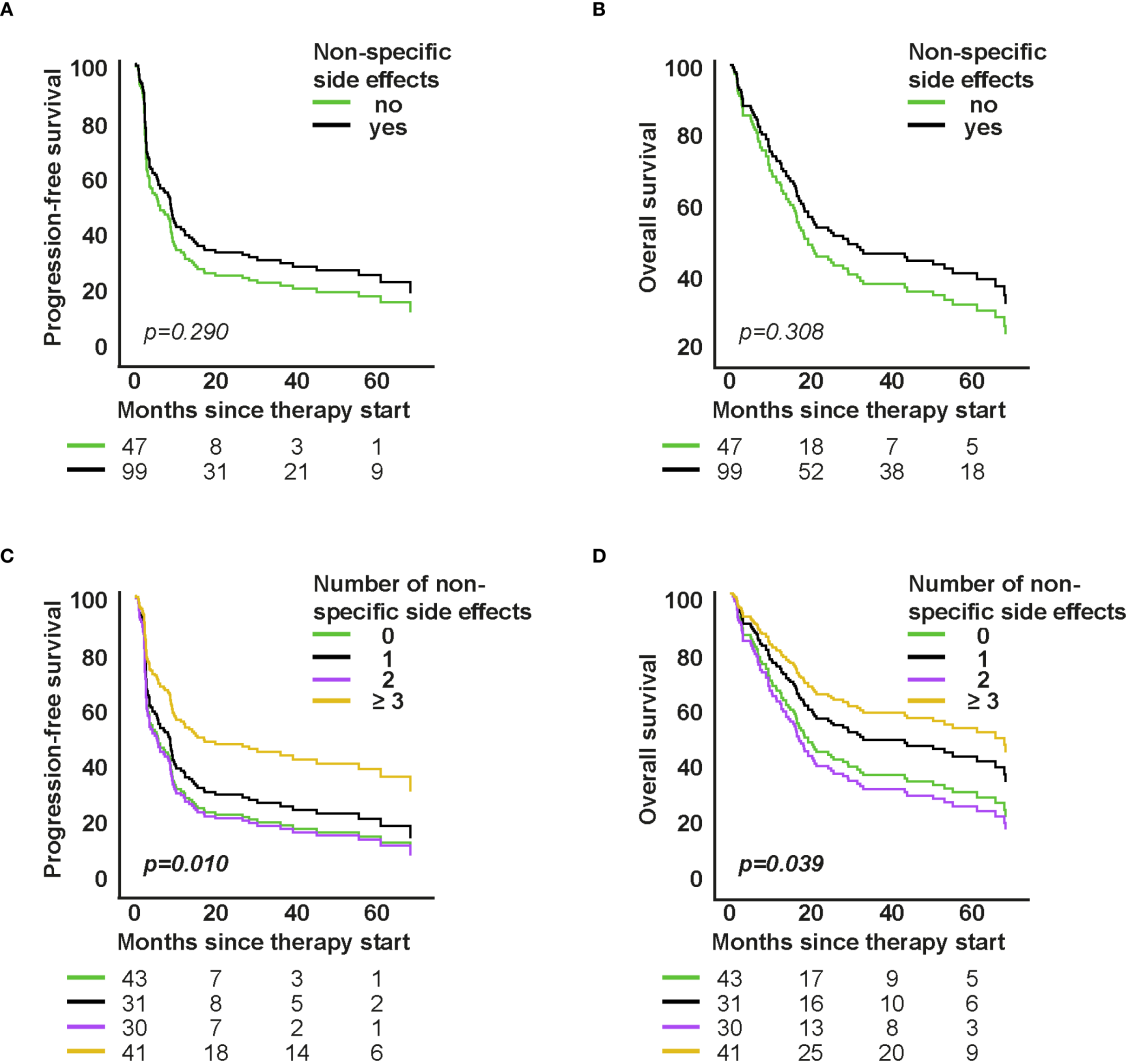
Association of non-specific symptoms with survival in first-line therapy. **A–D**: Cox proportional hazards models for PFS/OS by presence and number of non-specific symptoms. Curves show model-based survival functions; HRs (95% CI) and p-values are from the Wald test of the model coefficients.

Progression-free survival (PFS) was defined as the time from initiation of systemic therapy to the date of documented disease progression or last patient contact (censored). Overall survival (OS) was defined as the time from therapy initiation to death from any cause or last patient contact (censored). For all survival plots in the main manuscript (**Figures 2**–**4**) and in the **Supplementary Figures S2–S4**, survival functions were estimated using Cox proportional hazards models (IBM SPSS Statistics, version 29). Each model was fitted separately for the variable of interest (e.g., occurrence, number, or type of immune-related adverse events), and in some analyses, additional covariates were included as specified in the Results section. Plotted curves represent model-based survival functions from the fitted Cox models. Unless otherwise stated, hazard ratios (HR) and p-values presented in the figures are derived from the Wald test of the Cox model coefficients. This approach was chosen instead of unadjusted Kaplan–Meier curves to provide smoother survival estimates, which is particularly advantageous in smaller subgroups and when adjusting for covariates. Kaplan–Meier curves with corresponding log-rank tests for selected key comparisons were generated for reference and can be found in the **Supplementary Material** to allow direct visualization of unadjusted survival differences (**Supplementary Figure S5-S7** *)*. Receiver operating characteristic (ROC) curve analysis was applied to evaluate the prognostic accuracy of baseline blood parameters and their ratios in predicting PFS and OS. To determine the association between blood parameters and the occurrence of irAEs, logistic regression models were employed. Additionally, univariate and multivariate Cox proportional hazards regression analyses were conducted to assess the impact of baseline characteristics, treatment-related factors, irAEs, and blood-based markers on PFS and OS. Covariates in the multivariate Cox regression models included: age, gender, type of ICI therapy, BRAF mutation status, ECOG performance status, serum LDH, AJCCv8 stage (IIIC/D, M1a–M1d), and immunological factors, specifically the number and CTCAE toxicity grade of organ-specific irAEs. All statistical tests were two-sided, and a p-value < 0.05 was considered statistically significant.

## 3 Results

### 3.1 Patient and disease characteristics

Detailed baseline characteristics are summarized in **Table 1**. Among the 149 patients who received first-line immune checkpoint inhibitor (ICI) therapy, disease stages were distributed as follows (AJCC v8): 14.8% (n=22) were classified as unresectable stage IIIC/D, 10.1% (n=15) as stage IV M1a, 25.5% (n=38) as stage IV M1b, 26.8% (n=40) as stage IV M1c, and 22.8% (n=34) as stage IV M1d. Combination therapy with ipilimumab and nivolumab was administered in 24.8% (n=37) of patients, while 75.2% (n=112) received PD-1 inhibitor monotherapy. In the higher-line therapy cohort (n=82), 35.4% (n=29) were treated with combination therapy and 64.6% (n=53) with PD-1 monotherapy. Prior treatments in this group included BRAF- and MEK- inhibitors (68%), ipilimumab monotherapy (34%), dacarbazine (6.1%), nivolumab monotherapy (3.7%), pembrolizumab monotherapy (3.7%), T-VEC (1.2%), and prior ipilimumab plus nivolumab combination therapy (1.2%). Sex distribution was comparable between groups: 55.0% males versus 45.0% females in the first-line group, and 53.7% males and 46.3% females in the higher-line group. Median progression-free survival (PFS) was 8.5 months (95% CI: 6.19–10.83) in the first-line group and 12.5 months (95% CI: 1.73–23.37) in the higher-line group. Median overall survival (OS) was 27.3 months (95% CI: 10.07–44.47) and 26.3 months (95% CI: 2.18–50.52), respectively.

**Table 1:** Baseline characteristics according to therapy line.

Characteristics	First-line n=149 (100%)	Higher-line n=82 (100%)
Age		
≤65 years	60 (40.3)	61 (74.4)
>65 years	89 (59.7)	21 (25.6)
Sex		
Male	82 (55.0)	44 (53.7)
Female	67 (45.0)	38 (46.3)
Site of primary		
Cutaneous	110 (73.8)	65 (79.3)
Mucosal	18 (12.1)	5 (6.1)
Unknown primary	21 (14.1)	12 (14.6)
BRAF status		
V600 wildtype	108 (72.5)	26 (31.7)
V600 mutation	41 (27.5)	56 (68.3)
ECOG performance status		
0	83 (55.7)	52 (63.4)
1	53 (35.6)	24 (29.3)
≥2	13 (8.7)	6 (7.3)
Serum LDH		
Normal (≤ULN)	88 (59.1)	45 (54.9)
Elevated (>ULN)	71 (40.9)	37 (45.1)
Stage (AJCCv8)		
IIIC/D	22 (14.8)	2 (2.4)
IV M1a	15 (10.1)	6 (7.3)
IV M1b	38 (25.5)	20 (24.4)
IV M1c	40 (26.8)	23 (28.0)
IV M1d	34 (22.8)	31 (37.8)
First non-adjuvant therapy regimen		
CTLA-4+PD-1	37 (24.8)	29 (35.4)
PD-1	112 (75.2)	53 (64.6)
Best overall response		
CR	11 (7.4)	6 (7.3)
PR	40 (26.8)	32 (39.0)
SD	26 (17.4)	9 (11.0)
PD	60 (40.3)	17 (20.7)
Mixed response	6 (4.0)	9 (11.0)
Unknown	6 (4.0)	9 (11.0)
Therapy end reason		
Planned stop	21 (14.1)	15 (18.3)
Toxicity	23 (15.4)	18 (22.0)
Disease progression	77 (51.7)	40 (48.8)
Patient wish	12 (8.1)	2 (2.4)
Other	10 (6.7)	1 (1.2)
Ongoing	5 (3.4)	6 (7.3)
Lost to follow-up	1 (0.7)	
Progression		
No	42 (28.2)	29 (35.4)
Yes	107 (71.8)	53 (64.6)
Second-line therapy		
No	95 (63.8)	
Yes	54 (36.2)	
Death		
No	64 (43.0)	37 (45.1)
Yes	85 (57.0)	45 (54.9)
Progression-free survival		
Median in months (95% CI)	8.509 (6.191-10.827)	12.550 (1.734 – 23.367)
Overall survival		
Median in months (95% CI)	27.269 (10.072-44.466)	26.349 (2.179 – 50.519)

### 3.2 Immune-related adverse events

#### 3.2.1 Descriptive overview

Organ-specific and non-specific irAEs occurred frequently across the study cohort during ICI therapy. Gastrointestinal and cutaneous toxicities were the most frequent organ-specific events (**Figure 2A**). We analyzed vitiligo separately from other cutaneous irAEs because of its specific clinical relevance in melanoma, where vitiligo is often considered a surrogate marker of response. This separation allowed us to better capture its distinct prognostic significance. Gastrointestinal adverse events were coded according to CTCAE as colitis. Clinically, patients usually presented with diarrhea as the leading symptom. When colonoscopy confirmed colonic inflammation, the event was coded and graded as colitis; thus, diarrhea was not documented separately in order to avoid double counting, but consistently captured within the CTCAE category colitis. GI toxicities accounted for 29.7% of irAEs in first-line and 29.1% in higher-line therapy; cutaneous irAEs followed (26.6% and 21.8%, respectively). Among the thyroid cases, 3 presented with primary hyperthyroidism and 17 with hypothyroidism; in several patients, hyperthyroidism initially occurred and subsequently evolved into hypothyroidism. In the higher-line cohort, 16.4% had endocrine irAEs, with 22% hypophysitis (n=2) and 78% thyroiditis (n=7). Within these thyroid events, 1 patient developed primary hyperthyroidism, while 6 patients initially presented with hyperthyroidism that later evolved into hypothyroidism. Pulmonary irAEs accounted for 7.0% in first-line and 12.7% in the higher-line cohort, while rheumatologic irAEs were 4.7% and 7.3% respectively. Other irAEs (hematologic, neurologic, nephrologic, cardiovascular) each represented <5%. Non-specific symptoms were reported in 67% of patients during first-line and 72% during higher-line therapy (**Supplementary Figure S1**). In the first-line cohort, the most frequent of these symptoms were fatigue (23.0%), itch (14.8%), nausea (9.9%), loss of appetite (18.9%), and dry mouth (5.35%). In the higher-line cohort, fatigue (19.0%), itch (18.25%), and nausea (12.7%) were again most common, followed by loss of appetite (9.5%) and dry mouth (7.95%). Symptoms with an individual incidence below 5% were grouped into the category “Other,” which included dizziness, shortness of breath, hearing loss, hair loss, taste and smell disturbances, mucus production, vomiting, headache, fever, chills, flushing, numbness, abdominal pain, and constipation.

#### 3.2.2 Occurrence of organ-specific irAEs

In both therapy cohorts, the occurrence of organ-specific irAEs was significantly associated with improved PFS and OS (**Figures 2B, C** and **Supplementary Figures S2B**, **S2C**). In the first-line group, patients with irAEs (52.3%) had significantly longer PFS (median 9.96 vs. 5.09 months; p=0.012) and numerically longer OS (42.88 vs. 17.12 months; p=0.157) (**Figures 2B, C**). In the higher-line group, patients with irAEs (47.6%) showed also significantly longer PFS and OS (both p < 0.001) (**Supplementary Figures S2B**, **S2C**). Median OS could not be calculated due to censoring (64.1%), but mean OS was substantially longer (63.94 vs. 11.50 months).

#### 3.2.3 Number and severity of organ-specific irAEs

In the first-line therapy group, Cox proportional hazards analyses revealed a significant association between the number of organ-specific irAEs and improved outcomes. Patients with ≥2 irAEs had significantly longer progression-free survival (PFS) compared to those without irAEs (HR=0.494, 95% CI: 0.295–0.827; p=0.007), and also showed a reduced risk of death (OS: HR=0.531, 95% CI: 0.290–0.972; p=0.040) (**Figures 2D, E**). The severity of irAEs also impacted prognosis. Patients with mild to moderate irAEs (CTCAE grades I–III) showed longer PFS (HR=0.546, 95% CI: 0.365–0.817; p=0.003) and OS (HR=0.612, 95% CI: 0.389–0.964; p=0.034) compared to patients without organ-specific irAEs (**Figures 2F, G**). In contrast, high-grade irAEs (grades IV–V) were associated with significantly shorter OS (HR=2.552, 95% CI: 1.195–5.447; p=0.015), with a total of 9 patients experiencing CTCAE grade IV irAEs, and 2 patients experiencing fatal events (CTCAE grade V).

Similar patterns were observed in the higher-line therapy group. Patients with one irAE had a significantly reduced risk of progression (PFS: HR=0.296, 95% CI: 0.152–0.577; p < 0.001) and death (OS: HR=0.256, 95% CI: 0.117–0.560; p < 0.001) compared to patients with no irAEs. For patients with ≥2 irAEs, the trend was similar but did not reach statistical significance (PFS: HR=0.552, p=0.132; OS: HR=0.486, p=0.107). Regarding severity, 6 patients in the higher-line group experienced CTCAE grade IV irAEs and no patient died due to treatment-related toxicity (grade V), whereas mild to moderate irAEs (grades I–III) were again associated with better outcomes than higher ones (PFS: HR=0.375, 95% CI: 0.208–0.678; p=0.001; OS: HR=0.333, 95% CI: 0.171–0.651; p=0.001) (**Supplementary Figures S2F, S2G**).

#### 3.2.4 Impact of type of organ-specific irAEs on survival

Cutaneous irAEs were associated with a favorable trend toward improved PFS and OS (p=0.065), especially in the higher-line group. Vitiligo (n=10) significantly correlated with improved OS (HR=0.230; 95% CI: 0.057-0.936; p=0.040) and showed a trend for better PFS (HR=0.417; 95% CI: 0.169-1.026; p=0.057). Endocrine irAEs showed a trend for improved OS in higher-line therapy (p=0.043), whereas gastrointestinal irAEs showed no significant survival association (**Figure 3**; **Supplementary Figure S3**).

#### 3.2.5 Non-specific side effects

The occurrence of non-specific symptoms was not associated with survival in the first-line group (PFS: HR=0.801, 95% CI: 0.532–1.208; p=0.290; OS: HR=0.790, 95% CI: 0.501–1.243; p=0.308, **Figures 4A, B**), but was significantly associated with better outcomes in higher-line therapy (PFS: HR=0.451, 95% CI: 0.249–0.818; p=0.009; OS: HR=0.471, 95% CI: 0.249–0.891; p=0.021, **Supplementary Figures S4A, S4B**).

Further analyses revealed that patients experiencing ≥3 non-specific symptoms lived significantly longer. In the first-line group, this was associated with improved PFS (HR=0.505, 95% CI: 0.300–0.850; p=0.010) and OS (HR=0.538, 95% CI: 0.299–0.970; p=0.039; **Figures 4C, D**). Similar findings were observed in the higher-line group (PFS: HR=0.368, p=0.005; OS: HR=0.371, p=0.011; **Supplementary Figures S4C, S4D**), both findings suggesting that heightened systemic immune activity is associated with a better anti-tumoral response.

### 3.3 Systemic steroids and immunosuppressants

Corticosteroids were administered to 32.2% (first-line) and 29.3% (higher-line) of patients for irAE management. Seventeen first-line patients (11.4%) received corticosteroids before therapy for brain metastases. Of these, five patients later required additional corticosteroids for the management of treatment-related irAEs.

Among patients who received steroids during therapy for irAE management (n=56), prednisolone was the most commonly used agent. Clinical outcomes in this subgroup included partial response (PR) in 32 patients, stable disease (SD) in 7, and progressive disease (PD) in 14, indicating a favorable overall disease control rate despite concurrent immunosuppression. While corticosteroid use during therapy was not connected with poorer outcomes (PFS: p=0.221; OS: p=0.293), pre-treatment corticosteroids correlated with worse outcomes, likely reflecting disease severity. In this subgroup, 7 of 13 patients (53.8%) experienced progressive disease, 3 (23.1%) had partial response, 1 (7.7%) achieved a complete response, 1 (7.7%) had a mixed response, and 1 (7.7%) was not evaluable due to incomplete follow-up.

Four patients required second-line immunosuppression (e.g., infliximab (n=3) and mycophenolate mofetil (n=1)). Among infliximab-treated patients, clinical responses varied: one experienced PD, one achieved PR, and one had SD. The patient receiving mycophenolate mofetil achieved a PR, but no definitive survival patterns emerged due to small sample size.

### 3.4 Blood values

Baseline hematologic and serological parameters, including neutrophils, lymphocytes, eosinophils, monocytes, platelets, CRP, LDH, S100, and derived indices such as the neutrophil-to-lymphocyte ratio (NLR), lymphocyte-to-monocyte ratio (LMR), and platelet-to-lymphocyte ratio (PLR), were assessed prior to treatment initiation. In the first-line cohort, univariate analyses (**Table 2**) demonstrated that elevated neutrophil counts (HR 1.394; p=0.001), CRP levels (HR 1.494; p < 0.001) and NLR (HR 1.306; p=0.001) were significantly associated with shorter progression-free survival (PFS) and overall survival (OS), while higher relative lymphocyte counts (HR 0.698; p=0.001) and LMR values (HR 0.769; p=0.017) correlated with better survival. A higher eosinophil count was positively associated with both the occurrence of irAEs (OR 1.698; p=0.005) and improved overall survival (HR 0.779; p=0.040). In contrast, lower neutrophil counts (OR 0.711; p=0.050) and a lower NLR (OR 0.650; p=0.030) were also associated with an increased likelihood of developing irAEs, but unlike eosinophils, these markers were negatively associated with systemic inflammation and linked to better survival. Please note that a small number of baseline blood values were missing (range 2–13 across variables), which explains minor differences in sample size across biomarkers in **Table 2**.

**Table 2:** Exploratory univariate analysis of blood values (1^st^ line).

Blood Values	PFS HR (95%CI) p-value	OS HR (95%CI) p-value	irAEs OR (95%CI) p-value
Relative neutrophil count	**1.394 (1.141 – 1.703) 0.001**	**1.350 (1.086 – 1.679) 0.007**	**0.711 (0.505 – 1.000) 0.050**
Relative eosinophil count	0.852 (0.689 – 1.053) 0.138	**0.779 (0.614 – 0.988) 0.040**	**1.698 (1.175 – 2.453) 0.005**
Neutrophil-to-Lymphocyte Ratio (NLR)	**1.306 (1.120 – 1.523) 0.001**	**1.263 (1.053 – 1.515) 0.012**	**0.650 (0.440 – 0.960) 0.030**
C-reactive protein (CRP)	**1.494 (1.260 – 1.772) <0.001**	**1.310 (1.135 – 1.513) <0.001**	0.860 (0.601 – 1.230) 0.409
S100	**1.249 (1.065 – 1.464) 0.006**	**1.342 (1.119 – 1.609) 0.001**	0.829 (0.577 – 1.192) 0.313
Lactate Dehydrogenase (LDH)	**1.284 (1.107 – 1.489) 0.001**	**1.361 (1.165 – 1.589) <0.001**	0.953 (0.690 – 1.316) 0.769
Absolute leukocyte count	**1.237 (1.095 – 1.398) 0.001**	1.068 (0.934 – 1.221) 0.336	0.625 (0.375 – 1.040) 0.070
Absolute platelet count	**1.322 (1.112 – 1.571) 0.002**	**1.388 (1.148 – 1.679) 0.001**	0.925 (0.669 – 1.279) 0.636
Relative lymphocyte count	**0.698 (0.564 – 0.863) 0.001**	**0.736 (0.582 – 0.931) 0.010**	1.281 (0.915 – 1.792) 0.149
Relative monocyte count	0.938 (0.758 – 1.161) 0.558	0.888 (0.717 – 1.100) 0.276	1.385 (0.984 – 1.949) 0.062
Lymphocyte-to-Monocyte Ratio (LMR)	**0.769 (0.619 – 0.955) 0.017**	0.804 (0.635 – 1.018) 0.070	1.069 (0.768 – 1.487) 0.693
Platelet-to-Lymphocyte Ratio (PLR)	**1.316 (1.118 – 1.548) 0.001**	1.191 (1.000 – 1.419) 0.051	0.850 (0.607 – 1.189) 0.343

Sample size for first line therapy patients oscillates between n=149 and 136, with missing data for a small number of blood values (range 2–13 across variables). Hazard ratios in the first two columns were computed with univariate Cox regression analysis for progression-free survival and overall survival, respectively, while the odds ratios in the last column were computed using univariate logistic regression analysis to predict the probability of occurrence of irAEs during treatment. Continuous predictors were standardized. Bold values indicate statistically significant results (p < 0.05).

In the higher-line therapy cohort, the pattern was broadly similar but associations with irAEs were weaker (**Supplementary Table S1**). High baseline CRP (HR 1.697; p < 0.001), NLR (HR 1.402; p=0.020) and thrombocyte-to-lymphocyte ratio (HR 1.332; p=0.049) predicted shorter PFS and OS, while higher eosinophil counts remained associated with improved PFS and OS (HR 0.641 and 0.606; p=0.024 and 0.029, respectively). However, none of the blood parameters in the higher-line group showed a statistically significant association with irAE incidence.

To determine whether these associations were independent of clinical variables, all blood-based parameters were then included in multivariate Cox regression models, which adjusted for age, gender, ECOG status, BRAF mutation, LDH level, tumor stage, and irAE number and toxicity grade. In first-line therapy, CRP, neutrophil count, lymphocyte count, and LMR emerged as independent predictors of both PFS and OS (**Supplementary Table S2**). **Supplementary Table S3** reports results for the higher-line cohort.

Combining CRP with lymphocyte-based markers provided additional prognostic value. CRP plus lymphocyte count significantly improved PFS prediction (χ²=7.52, p=0.006 vs. χ²=4.67, p=0.031 for lymphocytes alone). Similar additive effects were observed when combining CRP with either neutrophils or LMR (**Supplementary Tables S4-S6**).

In the PFS models (**Table 3**), age >65 years and number of irAEs were associated with longer PFS, while severe irAEs predicted shorter PFS. Similarly, in the OS models (**Table 3**), BRAF mutation status and number of irAEs were linked to improved outcomes, whereas high-grade toxicity (compared to low to moderate toxicity) predicted reduced OS. Among all biomarkers, in addition to the number and toxicity grade of irAEs, CRP consistently demonstrated the strongest prognostic value, with elevated levels correlating with shorter PFS and OS. Additionally, a high lymphocyte count (Model 1) and LMR (Model 3) were predictive of longer progression-free survival (PFS), while a high neutrophil count (Model 2) was associated with unfavorable prognosis. Although the second biomarker in each OS model did not significantly improve model fit, the direction and magnitude of effects were consistent with findings in the PFS models in **Table 3**.

**Table 3:** Multivariate Cox-Regression model of blood values and side effects (1^st^ line).

Parameters (reference)	Model 1 HR (95%CI) p-value	Model 2 HR (95%CI) p-value	Model 3 HR (95%CI) p-value
Progression-free survival			
Gender (male)	1.311 (0.847 – 2.029) 0.225	1.296 (0.836 – 2.010) 0.247	1.360 (0.876 – 2.112) 0.171
Age (≤ 65 years)	**0.567 (0.344 – 0.934) 0.026**	**0.581 (0.352 – 0.958) 0.033**	**0.590 (0.360 – 0.968) 0.037**
BRAF (wildtype)	0.980 (0.598 – 1.605) 0.980	0.997 (0.609 – 1.632) 0.990	0.991 (0.602 – 1.633) 0.973
Tumor stage (IV M1d)			
III C/D	0.744 (0.369 – 1.502) 0.409	0.807 (0.393 – 1.656) 0.558	0.650 (0.323 – 1.307) 0.227
IV M1a	0.981 (0.448 – 2.144) 0.961	0.966 (0.441 – 2.119) 0.932	0.899 (0.415 – 1.947) 0.787
IV M1b	1.061 (0.581 – 1.937) 0.848	1.100 (0.595 – 2.031) 0.761	0.934 (0.513 – 1.702) 0.824
IV M1c	1.628 (0.884 – 2.999) 0.118	1.699 (0.914 – 3.158) 0.094	1.484 (0.813 – 2.708) 0.199
ECOG (0)			
0 vs. I	0.937 (0.598 – 1.470) 0.778	0.899 (0.573 – 1.409) 0.642	0.931 (0.594 – 1.460) 0.757
0 vs. >= II	0.597 (0.246 – 1.452) 0.255	0.604 (0.248 – 1.473) 0.268	0.547 (0.223 – 1.343) 0.188
LDH (normal)	1.405 (0.905 – 2.183) 0.130	1.441 (0.929 – 2.233) 0.102	1.481 (0.958 – 2.290) 0.077
Blood parameters			
CRP	**1.306 (1.059 – 1.610) 0.013**	**1.306 (1.055 – 1.617) 0.014**	**1.352 (1.108 – 1.650) 0.003**
Relative lymphocytes	**0.735 (0.586 – 0.922) 0.008**	–	**-**
Relative neutrophils		**1.288 (1.029 – 1.612) 0.027**	**-**
Lymphocyte-to-Monocyte Ratio (LMR)	**-**	–	**0.794 (0.637 – 0.989) 0.040**
Number of side effects	**0.549 (0.392 – 0.770) 0.001**	**0.553 (0.394 – 0.776) 0.001**	**0.525 (0.369 – 0.746) <0.001**
Toxicity Grade			
1 vs 0	0.781 (0.419 – 1.455) 0.436	0.778 (0.419 – 1.445) 0.426	0.728 (0.384 – 1.382) 0.332
1 vs 2	**4.445 (1.834 – 10.772) 0.001**	**4.451 (1.832 – 10.816) 0.001**	**3.790 (1.580 – 9.088) 0.003**
Overall survival			
Gender (male)	1.147 (0.702 – 1.873) 0.584	1.153 (0.704 – 1.887) 0.571	1.205 (0.739 – 1.966) 0.454
Age (≤ 65 years)	1.033 (0.559 – 1.906) 0.918	1.081 (0.590 – 1.981) 0.800	1.047 (0.568 – 1.927) 0.884
BRAF (wildtype)	**0.362 (0.182 – 0.724) 0.004**	**0.368 (0.185 – 0.732) 0.004**	**0.376 (0.189 – 0.748) 0.005**
Tumor stage (IV M1d)			
III C/D	0.583 (0.257 – 1.326) 0.198	0.651 (0.279 – 1.520) 0.322	0.507 (0.224 – 1.148) 0.103
IV M1a	0.411 (0.151 – 1.122) 0.083	0.424 (0.155 – 1.163) 0.095	0.381 (0.141 – 1.035) 0.058
IV M1b	0.686 (0.359 – 1.314) 0.256	0.720 (0.371 – 1.398) 0.332	0.614 (0.322 – 1.168) 0.137
IV M1c	1.221 (0.635 – 2.350) 0.550	1.294 (0.665 – 2.518) 0.447	1.116 (0.584 – 2.135) 0.740
ECOG (0)			
0 vs. I	0.875 (0.523 – 1.466) 0.613	0.846 (0.504 – 1.419) 0.527	0.886 (0.529 – 1.484) 0.645
0 vs. >= II	1.145 (0.479 – 2.734) 0.761	1.159 (0.485 – 2.767) 0.740	1.128 (0.472 – 2.694) 0.787
LDH (normal)	1.329 (0.820 – 2.154) 0.248	1.324 (0.816 – 2.147) 0.256	1.395 (0.867 – 2.246) 0.170
Blood parameters			
CRP	**1.264 (1.061 – 1.506) 0.009**	**1.263 (1.053 – 1.508) 0.010**	**1.286 (1.084 – 1.526) 0.004**
Relative lymphocyte	0.776 (0.598 – 1.008) 0.057	**-**	**-**
Relative neutrophils	**-**	1.256 (0.975 – 1.617) 0.078	**-**
Lymphocyte-to-Monocyte Ratio (LMR)	**-**	**-**	0.817 (0.631 – 1.058) 0.126
Number of side effects	**0.601 (0.417 – 0.868) 0.007**	**0.512 (0.423 – 0.884) 0.009**	**0.572 (0.391 – 0.838) 0.004**
Toxicity Grade			
1 vs 0	0.713 (0.356 – 1.429) 0.341	0.729 (0.364 – 1.456) 0.370	0.657 (0.323 – 1.337) 0.246
1 vs 2	**5.868 (2.261 – 15.227) <0.001**	**6.031 (2.292 – 15.866) <0.001**	**4.770 (1.885 – 12.070) 0.001**

All models computed with the same sample of n=142 patients and the same parameters except for the respective blood value added individually to each model. Continuous values such as blood parameters are standardized. PFS: Model 1 showed the best overall fit with the lowest −2 Log-Likelihood (−2LL=831.007) and Akaike Information Criterion (AIC=861.007), and the highest model chi-square (X²(15)=64.900, p <.001), compared to Model 2 (−2LL=833.552, AIC=863.552, X²(15)=62.565, p <.001) and Model 3 (−2LL=833.906, AIC=863.906, X²(15)=62.634, p <.001). OS: Model 1 demonstrated the best overall fit (−2LL=658.702, AIC=688.702, X²(15)=56.688, p <.001). Model 2 (−2LL=659.322, AIC=689.322, X²(15)=56.142, p <.001) and Model 3 (−2LL=659.956, AIC=689.956, X²(15)=56.281, p <.001) yielded comparable results, with only negligible differences in model fit (ΔAIC < 2). Bold values indicate statistically significant results (p < 0.05).

For progression-free survival (PFS), Model 1 provided the best overall fit, as indicated by the lowest −2 log-likelihood (−2LL=831.007) and the most favorable Akaike Information Criterion (AIC=861.007), compared to Models 2 and 3 (see **Table 3**). The same pattern held for OS (Model 1 AIC=688.702), although AIC differences between models were minimal (<3 points), indicating comparable model performance. Altogether, protective associations were seen for high lymphocyte count (Model 1) and LMR (Model 3), whereas increased neutrophil count (Model 2) consistently predicted poorer outcomes.

## 4 Discussion

Multiple studies have demonstrated a positive correlation between the occurrence of irAEs and improved progression-free survival (PFS) and overall survival (OS) in patients receiving immune checkpoint inhibitors (ICIs) (11–15). While this relationship is well established, key questions remain concerning the prognostic significance of irAE characteristics and the influence of pre-treatment inflammatory markers in context of irAEs, and whether these associations are consistent across different therapy lines.

Our study builds upon existing evidence by offering a therapy-line-specific analysis of irAEs and integrating both clinical and immunological variables into multivariate models. To our knowledge, this is the first study to systematically evaluate the combined prognostic relevance of irAE characteristics and baseline blood-based inflammatory markers in melanoma patients treated with PD-1-based immunotherapy. By simultaneously accounting for the number and CTCAE toxicity grade of organ-specific irAEs, as well as for systemic inflammatory profiles such as CRP, neutrophil and lymphocyte counts, we provide a more nuanced and clinically relevant understanding of how immune activation, systemic inflammation, and treatment outcomes interact in both first- and higher-line settings.

A central finding of our analysis is that the occurrence, number, and severity of irAEs are independently and also significantly associated with PFS and OS, regardless of therapy line. This reinforces the concept that irAEs may serve as surrogate markers for effective immune activation. However, we observed distinct differences between treatment lines: the prognostic impact of multiple irAEs was stronger in the first-line setting, potentially reflecting preserved immune competence and a more responsive tumor microenvironment. In contrast, patients undergoing higher-line therapy—often characterized by greater tumor burden, immune exhaustion from prior treatments, and more frequent corticosteroid or combination ICI use—exhibited attenuated immune responses and altered irAE profiles.

Our analysis also adds to existing literature by showing that patients who experienced multiple irAEs or endured moderate toxicities (CTCAE grade I–III) had particularly favorable outcomes, while high-grade irAEs (≥ CTCAE grade IV) were significantly associated with reduced overall survival in both therapy lines. This may result from intensified immunosuppressive interventions, such as infliximab or mycophenolate mofetil, or prolonged high-dose corticosteroid therapy, which, while effective in mitigating toxicity, could impair antitumor immunity (16, 17). These findings support the clinical relevance of not only capturing the presence of irAEs but also systematically quantifying their number and severity. This is in line with current management recommendations emphasizing timely recognition and graded intervention based on toxicity level (18).

Our analysis further underscores the prognostic value of pre-treatment inflammatory and hematological markers. Elevated CRP and neutrophil counts were associated with inferior outcomes, while higher lymphocyte-based markers, including relative lymphocyte count and LMR, were linked to longer PFS and OS. Among these, CRP emerged as the most robust and consistent independent predictor in multivariate models. These results highlight the importance of differentiating between general indicators of tumor-associated inflammation (e.g., CRP) and immune competence markers (e.g., lymphocyte count), which more directly reflect the patient’s capacity to mount an effective immune response.

Notably, while eosinophil counts were associated with survival and irAE development in univariate analyses, they did not retain independent prognostic significance in multivariate models, which included irAEs, suggesting that they correlate stronger with the occurrence of irAEs than with survival directly and therefore may play a secondary role in outcome prediction (19). In contrast, a low neutrophil-to-lymphocyte ratio (NLR) was associated with favorable survival outcomes, consistent with prior findings that link lower NLR to a more immunostimulatory environment (20–22).

Incorporating combinations of CRP and lymphocyte-derived parameters into multivariate models enhanced prognostic accuracy, especially for PFS in first-line therapy. Among the three evaluated models, the combination of CRP with relative lymphocyte count (Model 1) provided the best statistical fit. Although the differences between models were modest, the direction and effect sizes of the blood markers were consistent: higher lymphocyte and LMR values conferred benefit, whereas increased neutrophils predicted worse prognosis. These data suggest that integrating CRP with immune cell-based indices may be particularly useful in early-line treatment planning.

From a translational standpoint, the integration of clinical immune-related adverse events and baseline inflammatory biomarkers offers insight into the dynamic interplay between the innate and adaptive immune systems during immune checkpoint blockade. Immune-related toxicities such as dermatitis, colitis, or endocrinopathies likely reflect enhanced T-cell activation and systemic immune engagement (23). In contrast, elevated neutrophil counts and CRP levels may indicate tumor-associated inflammation, myeloid-derived suppressor cell (MDSC) expansion, or IL-6-driven immunosuppressive pathways (24–26). These opposing immune signatures may help explain why certain combinations of irAE profiles and blood-based markers confer distinct prognostic implications.

Furthermore, lymphocyte-based markers, such as LMR and absolute lymphocyte count, may serve as surrogate indicators of preserved immune competence, a prerequisite for effective tumor-specific responses. Our findings suggest that combining markers of immune toxicity (as indirect evidence of immune activation) with parameters reflecting inflammatory load and immune capacity could refine current response prediction models and inform personalized ICI strategies.

Future translational research should include immune profiling (e.g., cytokine signatures, T-cell repertoire analysis, and myeloid cell phenotyping) to mechanistically validate these associations and better define the immunological context of response and resistance in melanoma.

Nevertheless, this study has several limitations. First, it is a retrospective, single-center analysis, which may limit generalizability. Validation in independent, multicenter datasets is needed to strengthen external validity. The retrospective design may introduce selection bias, especially in patients who discontinued treatment early due to toxicity or progression. Second, a small number of baseline blood values were missing (range 2–13 across variables), resulting in a slightly reduced sample size in **Table 2** and potentially limiting statistical power for selected analyses. Also, the smaller sample size in the higher-line group restricts the interpretability of subgroup comparisons. In addition, treatment regimens were not stratified in detail, which may confound the observed associations between ICI type, irAE incidence, and outcomes. Finally, mechanistic explanations for the relationships between biomarkers, irAEs, and survival remain speculative. Future prospective studies incorporating longitudinal immune profiling and functional assays are essential to validate and mechanistically contextualize these findings.

In conclusion, this study highlights the prognostic value of the combination of immune-related adverse events and baseline inflammatory biomarkers in patients with metastatic melanoma treated with ICIs. The number and severity of irAEs emerged as independent predictors of survival, with moderate toxicity levels associated with the most favorable outcomes. CRP and lymphocyte-based markers provided additive prognostic information, particularly in first-line therapy and therefore should be applied in combination with irAEs. These findings underscore the complex but clinically relevant interplay between immune activation, systemic inflammation, and therapeutic response, and support a move toward more personalized approaches in immuno-oncology.

## Data Availability

The raw data supporting the conclusions of this article will be made available by the authors, without undue reservation.

## Glossary

AJCC: American Joint Committee on Cancer provides staging guidelines for cancer
CR: Complete Response disappearance of all signs of cancer in response to treatment
CRP: C-reactive Protein a marker of systemic inflammation in the blood
CTCAE: Common Terminology Criteria for Adverse Events a standard classification and severity grading scale for adverse effects
ECOG: Eastern Cooperative Oncology Group performance status scale to assess patient functional status
ICI: Immune Checkpoint Inhibitor drugs that block checkpoint proteins to enhance immune response against cancer
irAE: Immune-related Adverse Event side effects resulting from immune activation caused by ICIs
LDH: Lactate Dehydrogenase an enzyme that can indicate tissue damage or disease progression
LMR: Lymphocyte-to-Monocyte Ratio a derived index from blood counts used to reflect immune status
MDSC: Myeloid-Derived Suppressor Cells immune cells that suppress the anti-tumor immune response
NLR: Neutrophil-to-Lymphocyte Ratio a systemic inflammation marker associated with cancer prognosis
OR: Odds Ratio a statistic that quantifies the strength of the association between two events
OS: Overall Survival time from treatment initiation until death from any cause
PD: Progressive Disease cancer that is growing or spreading
PD-1: Programmed Cell Death Protein 1 a receptor targeted by ICIs
PD-L1: Programmed Death-Ligand 1 a ligand that binds PD-1 and downregulates immune responses
PFS: Progression-Free Survival time during and after treatment that a patient lives with the disease without it worsening
PLR: Platelet-to-Lymphocyte Ratio a prognostic marker derived from blood tests
PR: Partial Response a significant reduction in the size of tumors but not complete disappearance
ROC: Receiver Operating Characteristic a graphical plot used to show diagnostic ability of a binary classifier
SD: Stable Disease cancer that is neither decreasing nor increasing in extent or severity
