# Supplementary figures and images for: Combining immune-related adverse events and inflammatory profiles enhances prognostic accuracy in metastatic melanoma under PD-1-based therapy
Source: Front Immunol. 2025 Oct 1;16:1683533. doi: 10.3389/fimmu.2025.1683533 (PMC12521244; doi:10.3389/fimmu.2025.1683533)

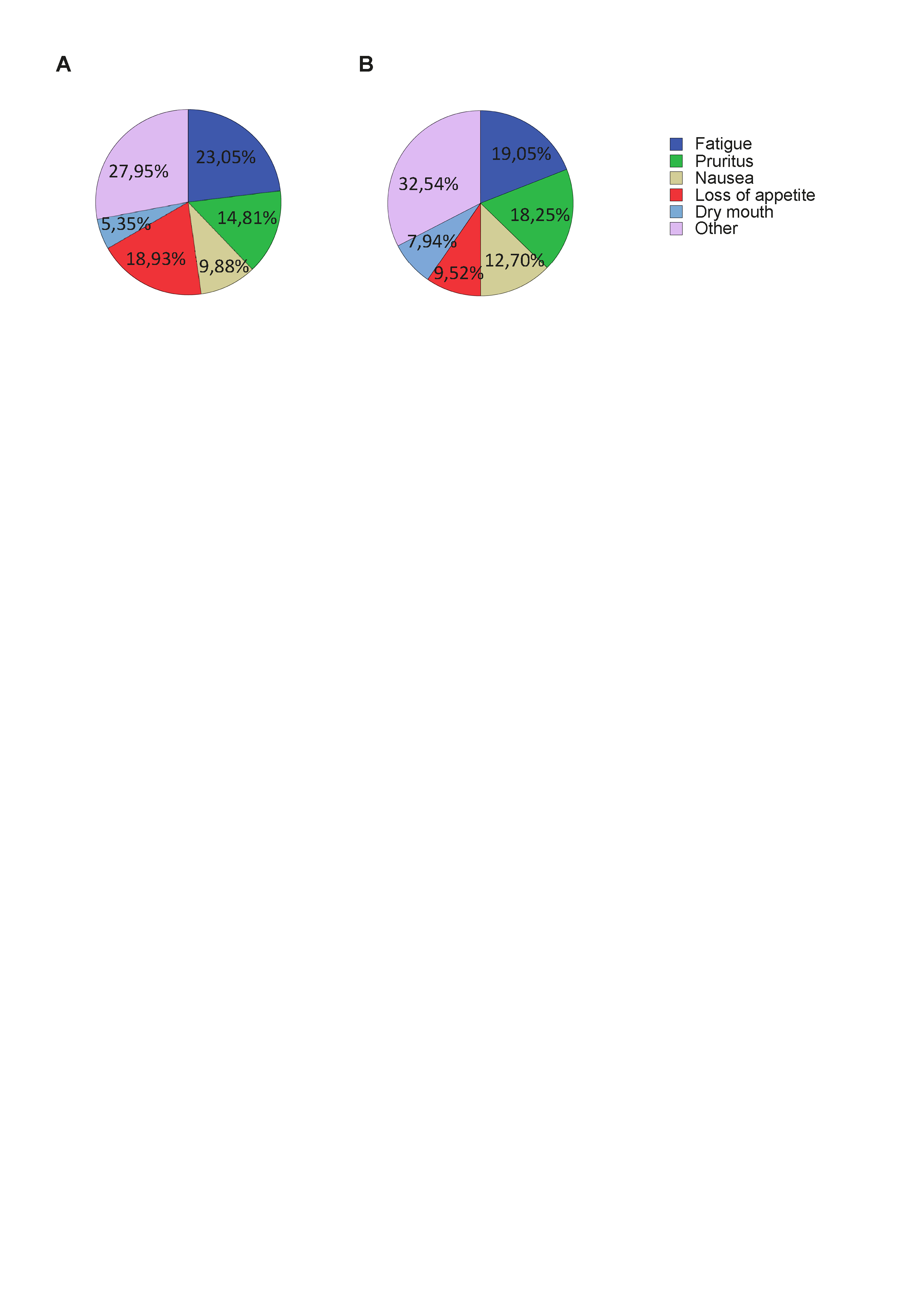

Supplement: Supplementary file 1 [file Image1.tif]

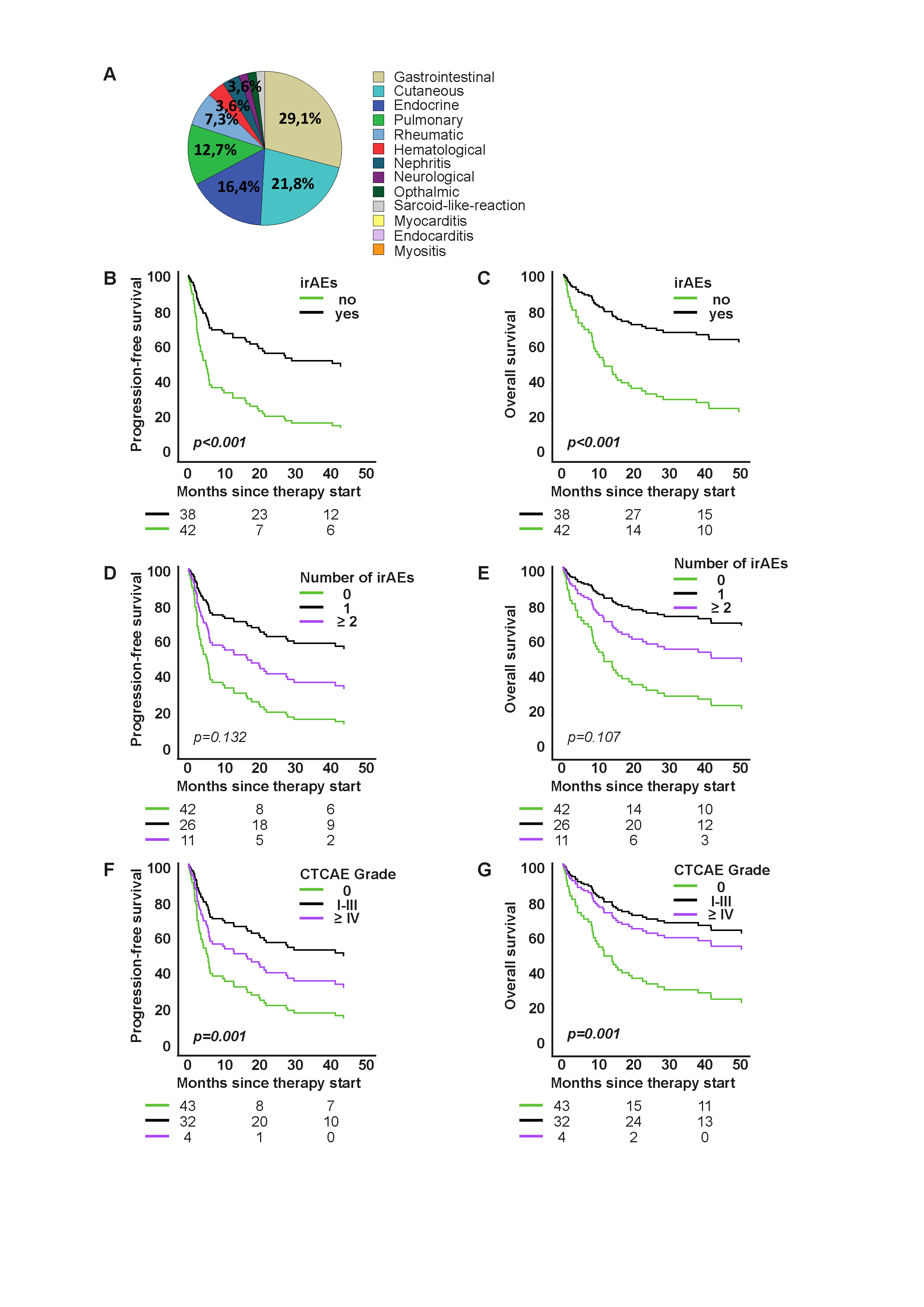

Supplement: Supplementary file 2 [file Image2.tif]

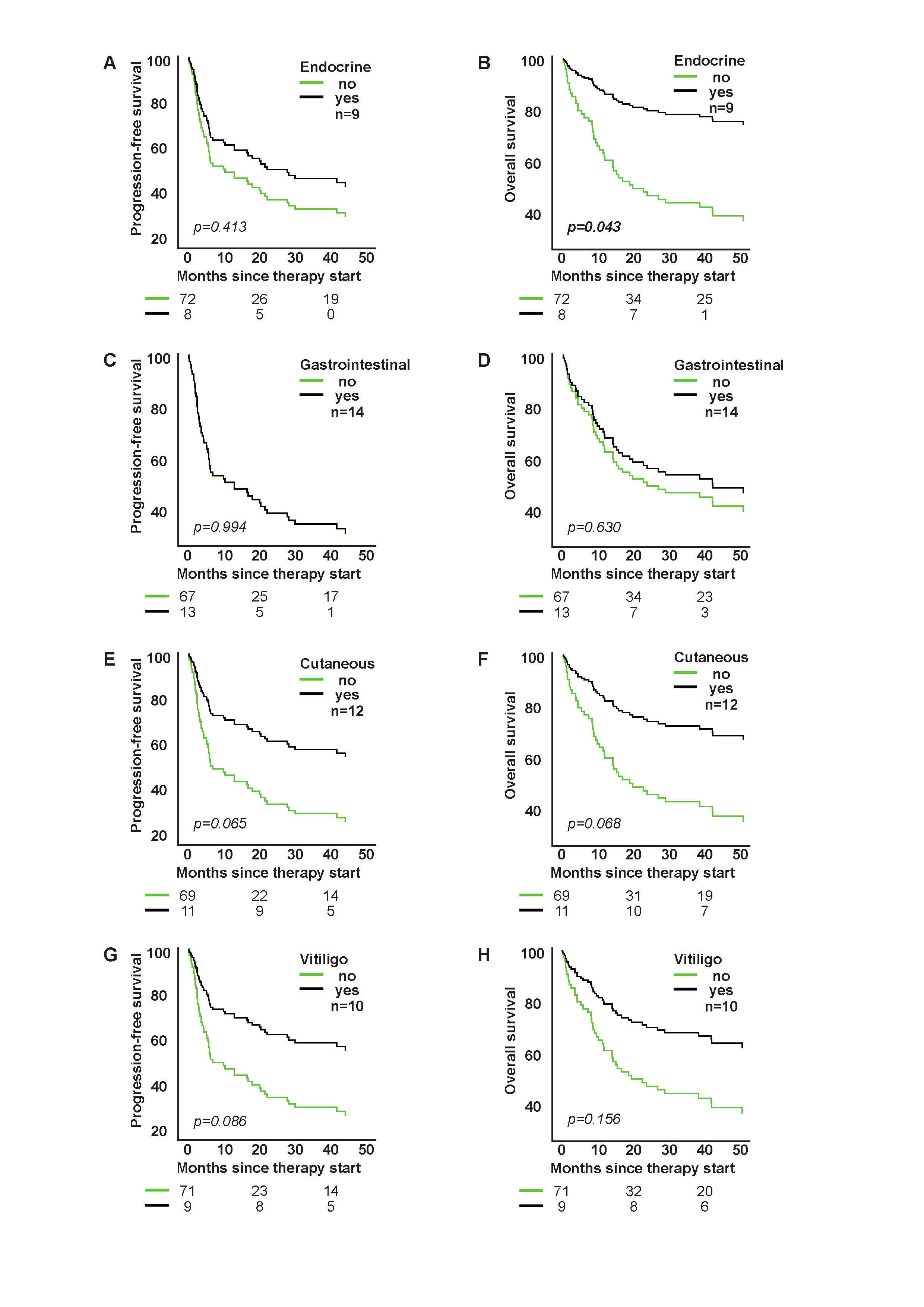

Supplement: Supplementary file 3 [file Image3.tif]

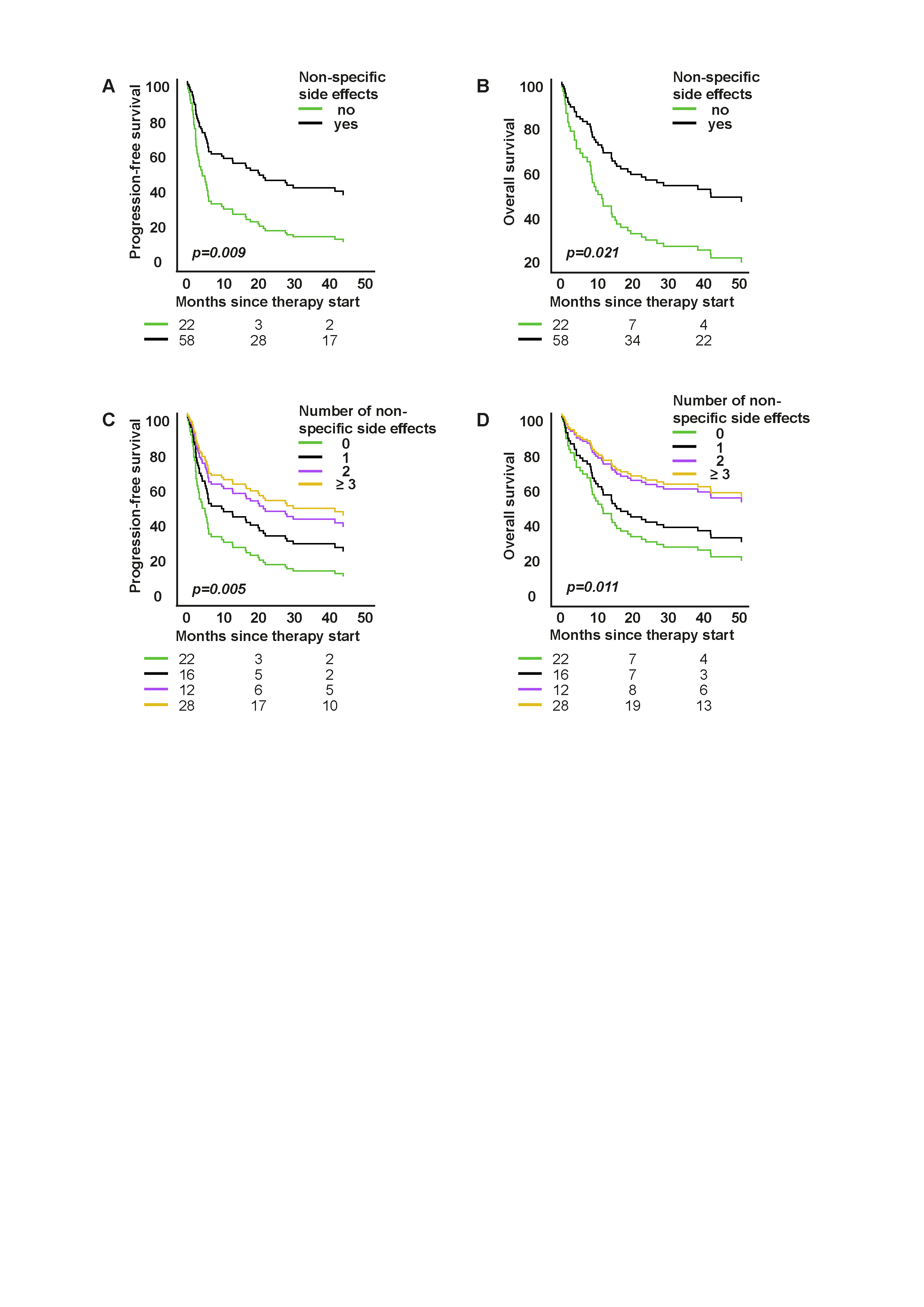

Supplement: Supplementary file 4 [file Image4.tif]

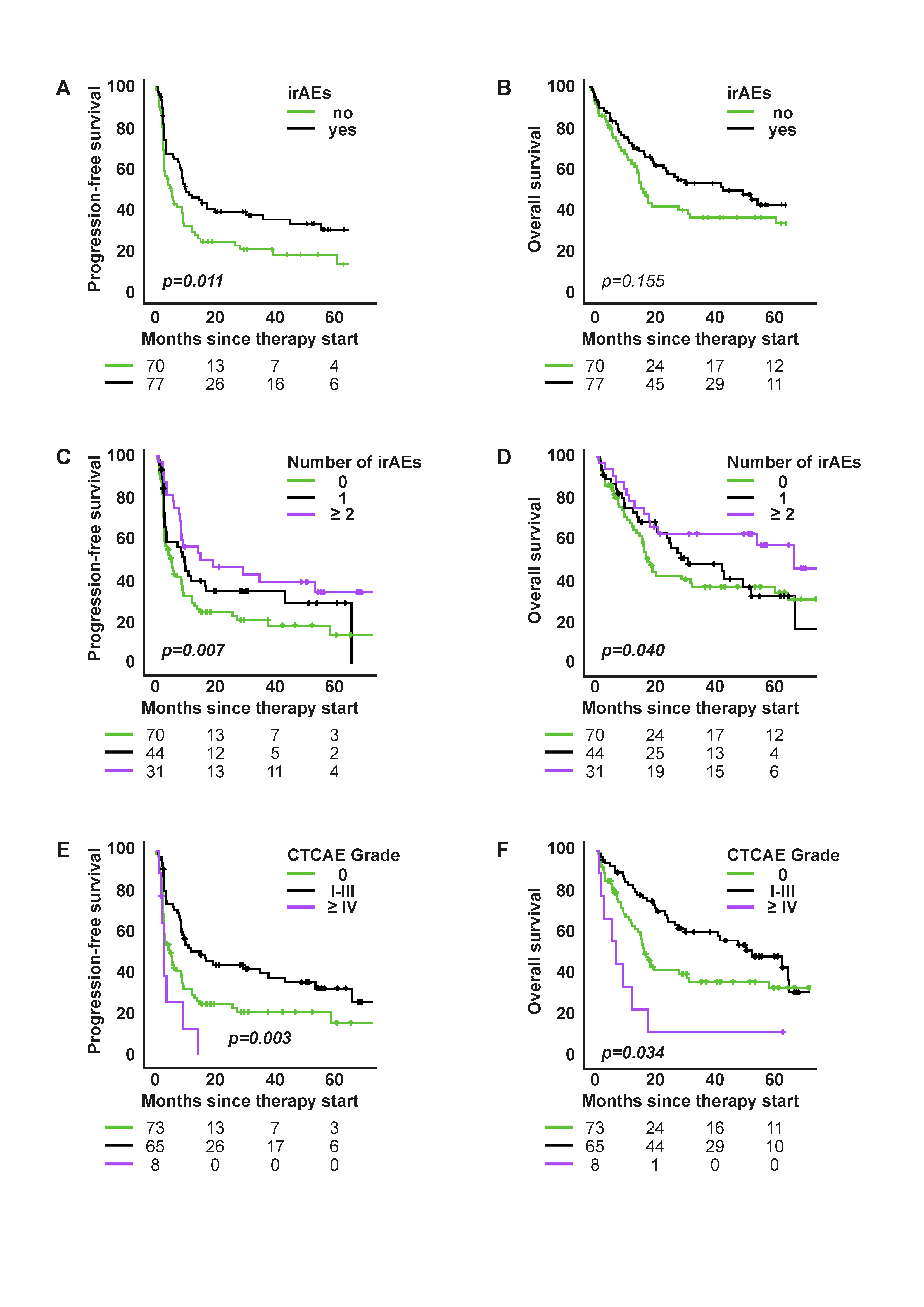

Supplement: Supplementary file 5 [file Image5.tif]

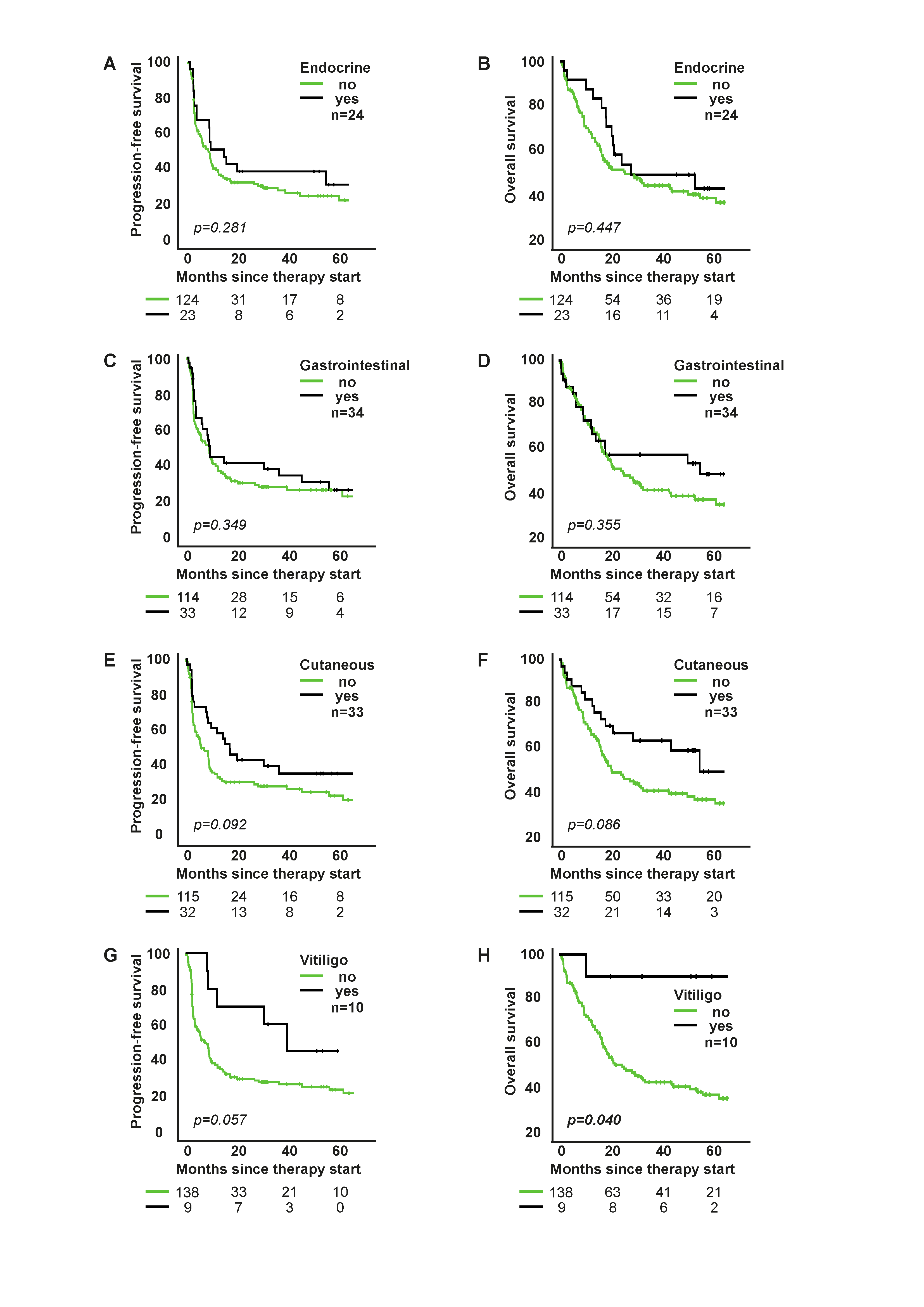

Supplement: Supplementary file 6 [file Image6.tif]

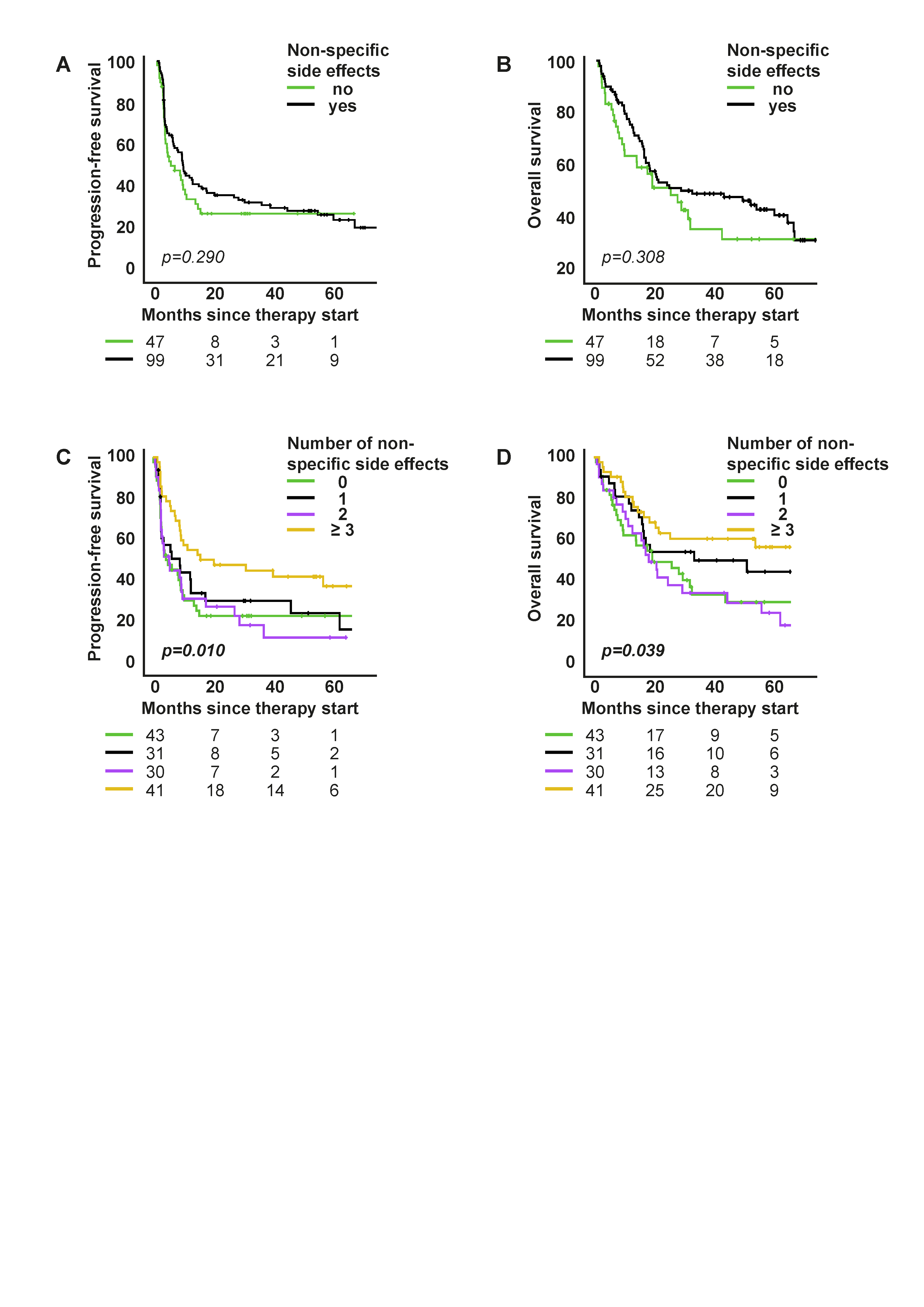

Supplement: Supplementary file 7 [file Image7.tif]
