# Supplementary material for: Combining immune-related adverse events and inflammatory profiles enhances prognostic accuracy in metastatic melanoma under PD-1-based therapy
Source: Front Immunol. 2025 Oct 1;16:1683533. doi: 10.3389/fimmu.2025.1683533 (PMC12521244; doi:10.3389/fimmu.2025.1683533)
Supplement: Supplementary file 8 [file SupplementaryFile1.docx]

**Figure S1.** **Reported non-specific side effects during first-line therapy (A) and higher-line therapy (B).** Side effects with an incidence of less than 5% were grouped into the category *Other*. This category included dizziness, shortness of breath, hearing impairment, hair loss, taste and smell disturbances, mucus production, vomiting, headache, fever, chills, flushing, numbness, abdominal pain, and constipation.

**Figure S2. The occurrence of organ-specific immune-related adverse events (irAEs) and survival in melanoma patients receiving immune checkpoint inhibitor therapy as a higher therapy line.** A: Distribution of organ-specific irAEs. B–C: Model-based survival curves from Cox proportional hazards models for PFS and OS by irAE occurrence. D–G: Cox models for PFS and OS by irAE number and toxicity grade. Curves show model-based survival functions from the respective Cox models. HRs and p-values are from the Wald test of the model coefficients.

**Figure S3. Impact of individual types of irAEs on survival in patients receiving immune checkpoint inhibitor therapy as a higher-line treatment.** A, C, E, and G: PFS; B, D, F, and H: OS. Each panel shows model-based survival functions from a separate Cox proportional hazards model for the respective irAE type. HRs (95% CI) and p-values are from the Wald test of the model coefficients.

**Figure S4. Association of non-specific irAEs with survival in melanoma patients receiving immune checkpoint inhibitor therapy as a higher-line treatment.** A–D: Cox proportional hazards models for PFS and OS by presence and number of non-specific irAEs. Curves show model-based survival functions; HRs (95% CI) and p-values are from the Wald test of the model coefficients.

**Figure S5. Association of organ-specific immune-related adverse events (irAEs) with survival under first-line immune checkpoint inhibitor therapy.** A-B: Kaplan-Meier curves for progression-free survival (PFS) and overall survival (OS) by irAE occurrence**.** C-F: Kaplan-Meier curves for PFS and OS by number of organ-specific irAEs and by toxicity grade. Log-rank *p*-values are reported; *p* < 0.05 was considered statistically significant. This figure serves as the unadjusted counterpart to the Cox model–based curves in Figure 2.

**Figure S6. Impact of individual organ-specific irAE types on survival in patients receiving first-line immune checkpoint inhibitor therapy.** A, C, E, G: PFS; B, D, F, H: OS. Log-rank *p*-values are reported; *p* < 0.05 was considered statistically significant. This figure serves as the unadjusted counterpart to the Cox model–based curves in Figure 3.

**Figure S7. Association of non-specific symptoms with survival in first-line therapy.** A–D: Kaplan–Meier survival curves with log-rank tests for occurence and number of non-specific side effects on PFS and OS. Log-rank *p*-values are reported; *p* < 0.05 was considered statistically significant. This figure serves as the unadjusted counterpart to the Cox model–based curves in Figure 4.
