# Supplementary material for: Combining immune-related adverse events and inflammatory profiles enhances prognostic accuracy in metastatic melanoma under PD-1-based therapy
Source: Front Immunol. 2025 Oct 1;16:1683533. doi: 10.3389/fimmu.2025.1683533 (PMC12521244; doi:10.3389/fimmu.2025.1683533)
Supplement: Supplementary file 9 [file SupplementaryFile2.docx]

| **Table S1**  **Exploratory univariate analysis of blood values for higher-line therapy patients** | | | |
| --- | --- | --- | --- |
|  | PFS  HR (95%CI) p-value | OS  HR (95%CI) p-value | irAEs OR (95%CI) p-value |
|  |  |  |  |
| Relative neutrophil count | 1.322 (0.955 – 1.830) 0.092 | 1.345 (0.947 – 1.911) 0.098 | 1.117 (0.704 – 1.773) 0.637 |
| Relative eosinophil count | **0.641 (0.435 – 0.944) 0.024** | **0.606 (0.386 – 0.951) 0.029** | 0.836 (0.522 – 1.340) 0.457 |
| Neutrophil-to-Lymphocyte Ratio (NLR) | **1.402 (1.054 – 1.865) 0.020** | **1.464 (1.087 – 1.973) 0.012** | 1.056 (0.667 – 1.673) 0.816 |
| C-reactive protein (CRP) | **1.697 (1.340 – 2.148) <0.001** | **1.701 (1.350 – 2.143) <0.001** | 0.910 (0.580 – 1.429) 0.683 |
| S100 | 0.888 (0.630 – 1.252) 0.498 | 0.934 (0.678 – 1.287) 0.675 | 1.951 (0.491 – 7.754) 0.343 |
| Lactate Dehydrogenase (LDH) | 1.294 (0.950 – 1.762) 0.102 | **1.455 (1.068 – 1.982) 0.017** | 0.842 (0.507 – 1.397) 0.506 |
| Absolute leukocyte count | 1.233 (0.928 – 1.639) 0.149 | 1.225 (0.919 – 1.633) 0.167 | 1.104 (0.713 – 1.709) 0.658 |
| Absolute thrombocyte count | 1.240 (0.941 – 1.634) 0.127 | 1.243 (0.948 – 1.628) 0.115 | 0.749 (0.467 – 1.200) 0.230 |
| Relative lymphocyte count | 0.809 (0.597 – 1.098) 0.174 | 0.780 (0.559 – 1.088) 0.144 | 0.953 (0.602 – 1.509) 0.838 |
| Relative monocyte count | 1.015 (0.752 – 1.369) 0.924 | 1.052 (0.764 – 1.449) 0.757 | 0.958 (0.614 – 1.496) 0.851 |
| Lymphocyte-to-Monocyte Ratio (LMR) | 0.787 (0.591 – 1.046) 0.099 | 0.738 (0.535 – 1.018) 0.065 | 0.947 (0.598 – 1.499) 0.815 |
| Thrombocyte-to-Lymphocyte Ratio (TLR) | **1.332 (1.001 – 1.772) 0.049** | **1.397 (1.030 – 1.895) 0.032** | 0.780 (0.473 – 1.284) 0.328 |
| **Note.** Sample size for higher line therapy patients oscillates between n=74 and 82, with a small number of missing blood values (range 0–8 across variables). Hazard ratios in the first two columns were computed with univariate Cox regressions for progression-free survival and overall survival, respectively, while the odds ratios in the last column were computed using univariate logistic regression analysis to predict the probability of occurrence of irAEs during treatment. Continuous predictors were standardized. | | | |

| **Table S2**  **Exploratory Multivariate Analyses of all blood parameters for first-line therapy patients** | | |
| --- | --- | --- |
|  | PFS  HR (95%CI) p-value | OS  HR (95%CI) p-value |
| Relative neutrophil count | **1.399 (1.132 – 1.729) 0.002** | **1.359 (1.067 – 1.731) 0.013** |
| Relative lymphocyte count | **0.961 (0.940 – 0.984) 0.001** | **0.966 (0.941 – 0.992) 0.011** |
| Lymphocyte-to-Monocyte Ratio (LMR) | **0.753 (0.607 – 0.932) 0.009** | **0.770 (0.598 – 0.990) 0.042** |
| C-reactive protein (CRP) | **1.432 (1.188 – 1.727) <0.001** | **1.332 (1.134 – 1.566) <0.001** |
| S100 | 1.175 (0.958 – 1.442) 0.122 | 1.230 (0.967 – 1.564) 0.092 |
| Absolute leukocyte count | 1.167 (0.993 – 1.373) 0.061 | 1.104 (0.911 – 1.338) 0.311 |
| Absolute platelet count | 1.182 (0.962 – 1.452) 0.113 | **1.339 (1.064 – 1.685) 0.013** |
| Relative monocyte count | 0.955 (0.771 – 1.184) 0.676 | 0.929 (0.745 – 1.159) 0.515 |
| Relative eosinophil count | 1.016 (0.812 – 1.272) 0.890 | 0.884 (0.672 – 1.164) 0.380 |
| Neutrophil-to-Lymphocyte Ratio (NLR) | **1.292 (1.074 – 1.553) 0.007** | 1.235 (0.999 – 1.527) 0.051 |
| Platelet-to-Lymphocyte Ratio (PLR) | **1.214 (1.014 – 1.454) 0.034** | 1.153 (0.938 – 1.416) 0.175 |
| **Note.** Sample size for first line therapy patients oscillates between n=149 and 136, with a small number of missing blood values (range 2–13 across variables). Hazard ratios in the first two columns were computed with multivariate Cox regression analysis for progression-free survival and overall survival, respectively, with a standard set of covariates and reference groups. Continuous predictors were standardized. The full multivariate cox regression contained the following covariates and references: Gender (male) + Age (≤ 65 years) + BRAF (wildtype) + Tumor stage (IV M1d) + ECOG (0) + LDH (≤ULN). | | |

| **Table S3**  **Multivariate Cox-Regression models of all other blood values on PFS and OS for higher-line therapy patients** | | | | | | |
| --- | --- | --- | --- | --- | --- | --- |
| Parameters  (reference) | PFS  HR (95%CI) p-value | | OS  HR (95%CI) p-value | |  | |
| S100B | | 0.993 (0.848 – 1.163). p=0.930 | | 0.084 (0.961-1.876). p=0.084 | |  |
| C-reactive protein (CRP) | | 1.007 (0.999-1.015). p=0.080 | | 1.007 (0.999-1.016). p=0.080 | |  |
| Absolute leukocyte count | | **1.140 (1.007-1.290). p=0.038** | | **1.177 (1.037-1.337). p=0.012** | |  |
| Absolute thrombocyte count | | 1.002 (0.998-1.006). p=0.336 | | 1.002 (0.998-1.006). p=0.276 | |  |
| Relative lymphocyte count | | 0.974 (0.938-1.012). p=0.180 | | 0.970 (0.931-1.010). p=0.138 | |  |
| Relative monocyte cout | | 0.931 (0.833-1.039). p=0.202 | | 0.920 (0.811-1.044). p=0.196 | |  |
| Lymphocyte-to-Monocyte Ratio (LMR) | | 0.819 (0.603-1.112). p=0.201 | | 0.778 (0.542-1.115). p=0.171 | |  |
| Thrombocyte-to-Lymphocyte Ratio (TLR) | | 1.197 (0.917-1.563). p=0.187 | | 1.210 (0.907-1.616). p=0.195 | |  |
|  | |  | |  | |  |

**Table S4**

**Crosstable for Multivariate Combinations on PFS for first-line therapy patients**

|  | (1) | (2) | (3) | (4) | (5) | (6) |
| --- | --- | --- | --- | --- | --- | --- |
| (1) C-reactive protein (CRP) | - | **0.006** | **0.026** | 0.079 | **0.032** | 0.156 |
| (2) Relative lymphocyte count | **0.031** | - | 0.794 | 0.936 | 0.701 | 0.916 |
| (3) Relative neutrophil count | **0.034** | 0.118 | - | 0.893 | 0.396 | 0.555 |
| (4) Neutrophil-to-Lymphocyte Ratio (NLR) | **0.015** | **0.018** | 0.071 | - | 0.087 | 0.617 |
| (5) Lymphocyte-to-Monocyte Ratio (LMR) | **0.011** | **0.029** | 0.085 | 0.165 | - | 0.381 |
| (6) Thrombocyte-to-Lymphocyte Ratio (TLR) | **0.007** | **0.004** | **0.013** | 0.093 | **0.037** | - |
| **Note**. The combination is tested in a multivariate cox regression with a standard set of covariates and adding a combination of blood values. The p-value in each cell indicates the improvement in model performance from a Chi-square test by adding the blood value of the column to a model which already contains the blood value of the row. The values between the diagonals vary based on the order of parameters when testing he models. Blood parameters without multivariate significance were not tested in combination with other blood parameters. | | | | | | |

**Table S5**

**Crosstable for Multivariate Combinations on OS for first-line therapy patients**

|  | (1) | (2) | (3) | (4) | (5) |
| --- | --- | --- | --- | --- | --- |
| (1) C-reactive protein (CRP) | - | 0.061 | 0.053 | 0.077 | 0.114 |
| (2) Absolute thrombocyte count | **0.013** | - | **0.023-** | **0.036-** | **0.045-** |
| (3) Relative lymphocyte count | **0.022** | 0.056 | - | 0.759 | 0.914 |
| (4) Relative neutrophil count | **0.024** | 0.066 | 0.440 | - | 0.406 |
| (5) Lymphocyte-to-Monocyte Ratio (LMR) | **0.013** | **0.030** | 0.135 | 0.121 | - |
| **Note**. The combination is tested in a multivariate cox regression with a standard set of covariates and adding a combination of blood values. The p-value in each cell indicates the improvement in model performance from a Chi-square test by adding the blood value of the column to a model which already contains the blood value of the row. The values between the diagonals vary based on the order of parameters when testing he models. Blood parameters without multivariate significance were not tested in combination with other blood parameters. | | | | | |

**Table S6**

**Combination of Blood parameters for Multivariate Cox regression for first-line therapy patients**

| Parameters A, B | A+B model HRs  HR (95%CI) p-value | Improvement over A | Improvement over B |
| --- | --- | --- | --- |
| **PFS** |  |  |  |
| A: C-reactive protein (CRP)  B: Relative lymphocyte count | A: 1.306 (1.059 – 1.610) 0.013  B: 0.735 (0.586 – 0.922) 0.008 | X2 = 7.519 **p=0.006** | X2 = 4.670 **p=0.031** |
| A: C-reactive protein (CRP)  B: Relative neutrophil count | A: 1.306 (1.055 – 1.617) 0.014  B: 1.288 (1.029 – 1.612) 0.027 | X2 = 4.974 **p=0.026** | X2 = 4.504 **p=0.034** |
| A: C-reactive protein (CRP)  B: Neutrophil-to-Lymphocyte Ratio (NLR) | A: 1.346 (1.096 – 1.652) 0.005  B: 1.201 (0.986 – 1.461) 0.068 | X2 = 3.086 p=0.079 | X2 = 5.861 **p=0.015** |
| A: C-reactive protein (CRP)  B: Lymphocyte-to-Monocyte Ratio (LMR) | A: 1.352 (1.108 – 1.650) 0.003  B: 0.794 (0.637 – 0.989) 0.040 | X2 = 4.620 **p=0.032** | X2 = 6.397 **p=0.011** |
| A: C-reactive protein (CRP)  B: Thrombocyte-to-Lymphocyte Ratio (TLR) | A: 1.379 (1.133 – 1.678) 0.001  B: 1.154 (0.953 – 1.398) 0.141 | X2 = 2.017 p=0.156 | X2 = 7.370 **p=0.007** |
| **OS** |  |  |  |
| A: C-reactive protein (CRP)  B: Relative lymphocyte count | A: 1.264 (1.061 – 1.506) 0.009  B: 0.776 (0.598 – 1.008) 0.057 | X2 = 3.757 p=0.053 | X2 = 5.208 **p=0.022** |
| A: C-reactive protein (CRP)  B: Relative neutrophil count | A: 1.263 (1.058 – 1.508) 0.010  B: 1.256 (0.975 – 1.617) 0.078 | X2 = 3.136 p=0.077 | X2 = 5.091 **p=0.024** |
| A: C-reactive protein (CRP)  B: Neutrophil-to-Lymphocyte Ratio (NLR) | A: 1.297 (1.095 – 1.536) 0.003  B: 1.177 (0.942 – 1.470) 0.150 | X2 = 1.922 p=0.166 | X2 = 6.647 **p=0.010** |
| A: C-reactive protein (CRP)  B: Lymphocyte-to-Monocyte Ratio (LMR) | A: 1.286 (1.084 – 1.526) 0.004  B: 0.817 (0.631 – 1.058) 0.126 | X2 = 2.503 p=0.114 | X2 = 6.171 **p=0.013** |
| A: C-reactive protein (CRP)  B: Thrombocyte-to-Lymphocyte Ratio (TLR) | A: 1.314 (1.112 – 1.552) 0.001  B: 1.109 (0.895 – 1.375) 0.342 | X2 = 0.863 p=0.353 | X2 = 7.362 **p=0.007** |
